# Opening Pandora's Box: making biological discoveries through computational data exploration

**DOI:** 10.1186/1745-6150-2-29

**Published:** 2007-11-20

**Authors:** L Aravind

**Affiliations:** 1 National Center for Biotechnology Information, National Library of Medicine, National Institutes of Health, Bethesda, MD 20894, USA

## Editorial

Over two decades ago the first electronic databases which systematically collected protein and nucleic acid sequence and structure data came into existence. This period also saw the emergence of the first algorithms and their implementations to query these databases for sequence and structure similarities, to align sequences, and to identify of compositional features in sequences. A parallel advance was the integration of these disparate data collections into strongly interconnected databases, which included repositories of biomedical literature (e.g. Entrez). It would be no exaggeration to state that these computational developments played a role comparable to that of the polymerase chain reaction in the rise of modern molecular biology.

The essential philosophy of this new movement within biology – computational biology – has been the use of computational methods to explore repositories of biological information to make new scientific discoveries. An early example of the success of these methods was the identification of the helix-turn-helix domain as a determinant of DNA-protein interaction [1]. This allowed the prediction of diverse bacterial and eukaryotic transcription factors, and resulted in testable hypotheses regarding the functions of key developmental regulators and oncogenes [2,3]. Ever since, computational investigations have resulted in discovery of new protein domains and prediction of their biochemical roles [4], discovery of new RNAs [5], identification of subcellular targeting signals in proteins [6] and prediction of transcription factor binding sites [7]. Application of such methodologies has also been at the heart of genomics – being central to the interpretation of genome sequences. Most remarkably it has allowed us to reconstruct the biology of diverse life forms, such as the syphilis pathogen [8], the malarial parasite [9] or the diverse uncultivable microorganisms [10], which were never too amenable to classical experimentation. The successes of genomics have also spawned whole assemblies of new forms of high-throughput data. These include genome-scale collations of data pertaining to gene expression, protein-protein interactions, genetic interactions and intra-population genomic polymorphisms. By adding a new layer of contextual information to that contained in sequences and structures of biomolecules these new datasets greatly add to the power of the computational discovery process.

The principal idea behind announcing the Discovery Notes section of *Biology Direct *is to augment the process of discovery in light of the unprecedented accumulation of biological data. The articles submitted to this section aim to occupy a specific niche in the already rich menagerie of publications. Papers announcing the sequencing of a particular genome or a high-throughput genome-scale analysis hardly do justice to all that can be inferred from the data presented in them. Especially in the case of the high-profile scientific magazines with a space-crunch, much is relegated to supplementary material and may not necessarily hit the target audience. Likewise, comprehensive papers discussing the evolution of particular biological systems might contain many specific findings that are lost in the bulk of the article. In light of this, we feel that key findings that are likely to elucidate previously obscure issues, provide novel connections or spur experimental investigations in new and unexpected directions are best published separately as succinct publications. Such publications would do greater justice to such discoveries that might not require a full-scale paper by making the key findings more accessible to the relevant audience. The value of such publications can be easily discerned by considering exemplars from the field of protein sequence analysis. For example, the papers announcing the identification of the UBA domain [11], or the BRCT domain [12] or the PAS domain [13,14] have enormously benefited, respectively, the study of the ubiquitin system, DNA repair and the sensing of light/redox stimuli.

The goal of the new 'Discovery Notes' section of Biology Direct is to publish brief reports of specific discoveries made by computational analysis of nucleic acid and/or protein sequences, structures or other data, with novel observations and conclusions about the function, organization, or evolution of proteins, genes or genomes. In format it will be comparable to the now discontinued Protein Sequence Motifs column in *TiBS *[4], and the currently active Genome analysis section in *TIG *and the Discovery Note section of *Bioinformatics*. Beyond the two above-mentioned venues, there are hardly any regular venues for such publications. The above venues employ the conventional peer review model involving three or more anonymous referees providing reports to the authors and confidential recommendations to the editors. As result, the usual vagaries of the process and the concomitant delays affect these publications. Given the swell in the data, and the high significance of some of these computational discoveries, we believe that the research community would benefit both from an additional forum for such publications, as well as a modified system of peer-review that would allow greater speed and openness.

In the original spirit of *Biology Direct*, the Discovery Notes section will follow the open peer-review format. However, there will be some key differences in the peer review process for the Discovery Notes section relative to conventional articles:

• Only two Editorial Board members are required for review of the manuscript.

• Referees should be selected from the Editorial Board that is specifically set up for discovery notes.

Reviewers would primarily assess the validity of the findings in the article, and have a right to veto publication if the findings are incorrect or trivial. The reviewers need not provide full comments/details of revisions, but are welcome to do so whenever they deem it fit. If reviewers see no requirement for additional comments, their report would merely indicate support for the publication of the manuscript. If either or both referees veto publication, the author should consider the manuscript rejected.

Given the nature of the peer-review of these articles, we believe it should not tax the reviewer excessively and he/she could return a review relatively quickly (~2 weeks). All other aspects of the peer review process remain the same as for research articles submitted to *Biology Direct*. Central to the success of such a process is an Editorial Board with a strong track record in computational analysis of biological data. Approximately 40 excellent researchers have accepted our invitation to join the Editorial Board of Discovery Notes at *Biology Direct*. While such a starting base is encouraging, it is clear that the ultimate success of this section would depend on the authors submitting their interesting findings for publication. We do hope that this section provides a venue for fostering an active community of explorers seeking biological discoveries *in silico*.
